# Associations between ocular biometry and anthropometric measurements among Sudanese adults

**DOI:** 10.25122/jml-2024-0292

**Published:** 2024-09

**Authors:** Raghda Faisal Mutwaly

**Affiliations:** 1 Department of Optometry, College of Applied Medical Sciences, Qassim University, Buraydah, Saudi Arabia; 2 Department of Neurology of Vision, Faculty of Optometry and Visual Sciences, Al-Neelain University, Khartoum, Sudan

**Keywords:** ocular biometry, anthropometric parameters, axial length, anterior chamber depth, body mass index

## Abstract

Correlations between body parameters and ocular parameters are essential to emphasize the diagnosis and management of ocular and systemic diseases. This study aimed to assess the associations between ocular parameters and anthropometric parameters in adult Sudanese individuals. A cross-sectional hospital-based study was conducted with 250 young volunteers (250 eyes) at Al-Neelain University Eye Hospital from January to June 2019. Clinical examinations included demographic data, medical history, visual acuity assessment, refractive error, and anterior corneal power (ACP) measurement using an autorefkeratometer and ocular biometry via A-scan ultrasound. Anthropometric assessments included height (measured using a wall-mounted metric ruler), weight (measured with a digital scale), and body mass index (BMI), calculated as weight divided by height squared. Data analysis was performed using SPSS version 25. There were 64 (25.6%) men and 186 (74.4%) women. The mean age was 21.29 ± 1.18 years. The mean body height, weight, and BMI were 1.62 ± 0.07 m, 58.56 ± 11.93 kg, and 22.38 ± 4.80 kg/m^2^, respectively. The mean axial length (AL), ACP, anterior chamber depth (ACD), and vitreous depth (VD) were 22.81 ± 0.74 mm, 43.30 ± 1.40 D, 3.20 ± 0.33 mm, and 15.97 ± 0.67 mm, respectively. Body height was positively correlated with AL, ACD, and VD and negatively correlated with ACP (*P* < 0.001). Body weight was significantly positively correlated with AL and VD (*P* < 0.05). BMI was not correlated with any ocular parameters (*P* > 0.05). The study concluded that taller subjects had significantly longer axial lengths, deeper vitreous cavities, and flatter corneas. However, body weight was positively associated with axial length and vitreous depth.

## INTRODUCTION

Biometry is relevant to the measurement of living tissue in humans. Ocular biometry is important in the preoperative assessment of cataract surgery because it involves measuring the intraocular lens power, anterior corneal curvature, and axial lens of the eye. These ocular measurements can be influenced by genetics, race, and ethnicity [1]. Currently, obesity represents a major public health problem worldwide and is increasing in prevalence in most countries. According to the World Health Organization (WHO), more than 1.9 billion and 650 million people are overweight or obese worldwide, respectively [2,3]. The WHO developed a body mass index (BMI) classification system for identifying obesity. It is typically classified into three groups: underweight (BMI < 18.5 kg/m^2^), normal body weight (BMI: 18.50–24.99 kg/m^2^), and overweight (BMI ≥ 25 kg/m^2^) [4,5]. Obesity is a risk factor for many chronic systemic diseases, such as diabetes mellitus, hypertension, cardiovascular disease, and stroke. It also impacts the quality of life of humans [6].

However, obesity is associated with several ocular diseases, such as cataracts, diabetic retinopathy, age-related macular degeneration, and glaucoma. These diseases lead to a reduction in visual acuity and potential blindness in advanced cases [7-9]. In addition, the associations between ocular parameters and anthropometric parameters are essential in diagnosing and managing ocular and systemic diseases and detecting any abnormality in the early stages. Previous studies reported that ocular biometry is correlated with systemic biometric parameters such as sex, age, body weight, body height, and BMI [10,11]. These parameters and their correlations may differ from one area to another depending on the environment, race, body health, and socioeconomic state.

Compared with normal-weight individuals, underweight and overweight individuals may have alterations in body measurements [12,13]. Previous studies have demonstrated that demographic variables such as age, sex, and height are associated with various biometric body parameters. For instance, axial length (AL) has been shown to correlate positively with anterior chamber depth (ACD) and negatively with lens thickness (LT), with a negative relationship between LT, AL, and ACD [14]. In the Egyptian population, taller individuals tend to have longer ALs, but no significant association has been found between weight or BMI and any ocular parameters [2]. In contrast, other studies have reported a positive relationship between weight and AL, while BMI does not seem to correlate with ocular measurements. Additionally, age was positively correlated with ACP and LT and negatively correlated with height and ACD [15,16].

However, significant differences in ocular parameters between males and females have been observed in South Asia. Both height and weight were negatively correlated with corneal power, while BMI showed no association with ocular parameters except for AL [17-19]. In addition, the refractive state of the eye was significantly correlated with biometric body parameters. These relationships between ocular and anthropometric parameters are essential in diagnosing and managing ocular and systemic diseases [20, 21]. Interestingly, individuals with higher BMI tend to be more hypermetropic compared to those with lower BMI [22]. A study conducted in a Malaysian population further reported that changes in ocular dimensions, particularly in young adults, were associated with body height [23].

According to previous studies, there is a significant correlation between the severity of obesity and ocular parameters, as well as anthropometric parameters. No study has been conducted on the Sudanese population. Therefore, this study aimed to determine the correlation between ocular and anthropometric parameters in Sudanese adults. Additionally, this study aimed to provide clinicians with a basis for defining the clinical features of the patient’s body and eye.

## Material and Methods

A descriptive cross-sectional, hospital-based study was conducted on 250 healthy Sudanese volunteers (250 eyes) at Al-Neelain University Eye Hospital, Khartoum, Sudan, from January to June 2019. The study was approved by the Scientific Research Deanship of Al-Neelain University, and informed consent was obtained from all participants before clinical examinations. The inclusion criteria were young adult participants aged 20–25 years with healthy, emmetropic eyes (spherical equivalent ≤ 0.75 DS in the right eye), normal intraocular pressure (10–20 mmHg), and good visual acuity (6/6). Both genders were included. Exclusion criteria comprised a history of ocular or systemic diseases, intraocular surgery, eye trauma, mental retardation, or prior gastric sleeve surgery. Participants were selected using a convenience sampling method from university students.

Data collected during clinical examinations included demographic information (age and sex), medical history, unaided visual acuity (measured in Decimal notation), refractive error (in diopters), and anterior corneal curvature (in diopters) measured using an autoref-keratometer (Topcon RK-8900). Ocular parameters, including AL, ACD, and VD, were measured using A-scan ultrasound biometry (Nidek-4000). Anthropometric measurements included height (in meters), measured with a wall-mounted metric ruler, and weight (in kilograms), measured with a digital scale. BMI was calculated using the formula BMI = weight (kg) / height^2^ (m^2^). For accuracy, three measurements were taken for each variable, and the average was used. Ocular measurements were performed only on the right eye. Participants were classified as underweight (<18.5 kg/m^2^), normal weight (18.50–24.99 kg/m^2^), or overweight (≥25 kg/m^2^) according to the WHO international classification of obesity in adults.

Data were analyzed using IBM SPSS version 25. Descriptive statistics were generated, including frequencies, minimum and maximum values, means, and standard deviations. The means of study variables (ACP, AL, ACD, VD, height, weight, and BMI) were compared across groups using one-way ANOVA at a 95% confidence level. Statistical significance was set at *P* < 0.05.

## RESULTS

A total of 250 healthy Sudanese adults (250 eyes) participated in this study. Sixty-four (25.6%) were men, and 186 (74.4%) were women. The mean age of the participants was 21.29 ± 1.18 years (range: 20 to 24 years). The mean body height was 1.62 ± 0.07 m (range: 1.45 to 1.87 m), the mean body weight was 58.56 ± 11.93 kg (range: 32 to 1129 kg), and the mean BMI was 22.38 ± 4.80 kg/m^2^ (range: 14.20 to 60.60 kg/m^2^).

The mean AL was 22.81 ± 0.74 mm (range: 20.22 to 25.00 mm), the mean ACP was 43.30 ± 1.40 D (range: 40.38 to 49.62 D), the mean ACD was 3.20 ± 0.33 mm (range: 2.17 to 4.27 mm), and the mean VD was 15.97 ± 0.67 mm (range: 13.85 to 17.83 mm). Pearson’s sample correlation coefficient (r) test revealed no significant correlation between clinical parameters and age (*P* > 0.05). One-way ANOVA test showed significant differences between men and women in age (*P* = 0.42), height (*P* < 0.001), weight (*P* = 0.006), AL (*P* = 0.005), and ACP (*P* < 0.001). No significant difference was found in SE (*P* = 0.231), BMI (*P* = 0.071), ACD (*P* = 0.304), and VD (*P* = 0.141) (Table 1).

**Table 1:** Clinical parameters of the participants

Parameter	Total (*n* = 250)	Men (*n* = 64)	Women (*n* = 186)	*P* value^a^	*P* value^b^
	Mean ± SD (range)	Mean ± SD (range)	Mean ± SD (range)		
Age (years)	21.29 ± 1.18 20–24	21.55 ± 1.36 20–24	21.20 ± 1.11 20–24		0.042
SE (D)	0.38 ± 0.19 0.12–0.75	0.20 ± 0.26 0.00–0.50	0.35 ± 0.17 0.25–0.75	0.351	0.231
Height (m)	1.62 ± 0.07 1.45–1.87	1.68.90 ± 8.64 1.48–1.87	1.59.69 ± 6.08 1.45–1.74	0.241	<0.001
Weight (kg)	58.56 ± 11.93 32–129	62.06 ± 14.20 46–129	57.35 ± 10.82 32–90	0.224	0.006
BMI (kg/m^2^)	22.38 ± 4.80 14.20–60.60	21.44 ± 3.80 15.60–40.70	22.70 ± 5.08 14.20–60.60	0.803	0.071
AL (mm)	22.81 ± 0.74 20.22–25.00	23.03 ± 0.74 20.52–25	22.73 ± 0.72 20.67–24.90	0.739	0.005
ACD (mm)	3.20 ± 0.33 2.17–4.27	3.24 ± 0.29 2.75–4.27	3.19 ± 0.35 2.17–4.04	0.546	0.304
VD (mm)	15.97 ± 0.67 13.85–17.83	16.08 ± 0.69 13.85–17.62	15.94 ± 0.67 14.32–17.83	0.599	0.141
ACP (D)	43.30 ± 1.40 40.38–49.62	42.56 ± 0.97 40.50–44.63	43.56 ± 1.44 40.38–49.62	0.839	<0.001

SE, sphere equivalent; BMI, body mass index; AL, axial length; ACD, anterior chamber depth; VD, vitreous depth; ACP, anterior corneal power. ^a^ Pearson correlation test; ^b^ Analysis of variance (ANOVA).

In addition, the participants were classified into three groups: 166 (66.40%) had a normal weight, 39 (15.60%) were underweight, and 45 (18%) were overweight. One-way ANOVA revealed significant differences in weight (F = 128.22; *P* < 0.001) and BMI (F = 200.80.22; *P* < 0.001) between the study groups. However, there was no significant difference in other ocular or anthropometric parameters between the study groups (*P* > 0.05) (Table 2).

**Table 2:** Clinical parameters of the study groups

Parameter	Underweight (*n* = 39) (BMI<18.5 kg/m^2^)		Normal weight (*n* = 166) (BMI:18.50–24.99 kg/m^2^)		Overweight (*n* = 45) (BMI≥25k/m^2^)		ANOVAP value
	Mean ± SD	Range	Mean ± SD	Range	Mean ± SD	Range	
Height (m)	163.79 ± 8.55	150–181	161.75 ± 7.28	146–186	161.60 ± 9.45	145–187	0.322
Weight (kg)	47.02 ± 7.15	32–66	56.78 ± 6.57	42–74	75.11 ± 13.75	58–129	<0.001
BMI (kg/m^2^)	17.28 ± 1.09	14.2–18.4	21.58 ± 1.79	18.6–24.9	29.74 ± 6.06	25–60	<0.001
AL (mm)	22.70 ± 0.88	20.52–24.40	22.83 ± 0.65	21.44–25	22.82 ± 0.91	20.67–24.90	0.634
ACD (mm)	3.16 ± 0.35	2.17–3.90	3.22 ± 0.30	2.44–4.27	3.17 ± 0.44	2.18–4.04	0.532
VD (mm)	15.90 ± 0.75	13.85–17.50	15.97 ± 0.63	14.68–17.83	16.07 ± 0.74	14.66–17.83	0.500
ACP (D)	43.30 ± 1.33	40.88–46.36	43.27 ± 1.40	40.50–49.62	43.41 ± 1.51	40.38–46.80	0.819

BMI, body mass index; AL, axial length; ACD, anterior chamber depth; VD, vitreous depth; ACP, anterior corneal power.

**Figure 1:**
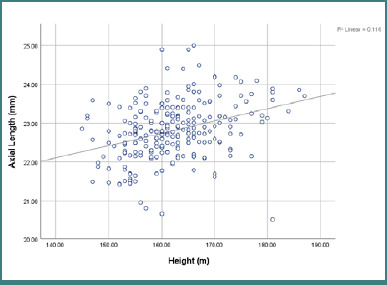
Correlations between axial length and height

Furthermore, the correlations between ocular and anthropometric parameters in the study population were examined via Pearson’s correlation test. Body height was positively correlated with body weight (r = 0.431; *P* < 0.001), AL, and VD (*P* < 0.001) and negatively correlated with ACPs (r = -0.287; *P* < 0.001). Additionally, it was not correlated with ACD or BMI (*P* > 0.05) (Figure 1).

However, body weight was significantly positively correlated with BMI (r = 0.721; *P* < 0.001), AL, and VD (*P* < 0.05) but not with ACD or ACP (*P* > 0.05). Moreover, BMI was strongly positively correlated with weight (r = 0.721; *P* < 0.001) but not with other clinical parameters (*P* > 0.05) (Table 3).

**Table 3:** Correlations between ocular parameters and anthropometric parameters

Parameter	Correlation	AL	ACD	VD	ACP
Body height (m)	*r*	0.340	0.092	0.317	-0.287
	*P*	<0.001	0.146	<0.001	<0.001
Body weight (kg)	*r*	0.161	-0.005	0.156	-0.070
	*P*	0.011	0.941	0.014	0.273
Body mass index (kg/m^2^)	*r*	-0.004	-0.065	0.069	0.097
	*P*	0.949	0.303	0.279	0.125

AL, axial length; ACD, anterior chamber depth; VD, vitreous depth; ACP, anterior corneal power.

**Figure 2:**
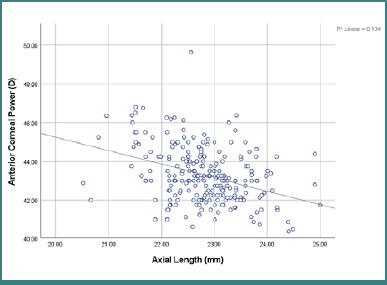
Correlations between axial length and anterior corneal power

In addition, Pearson’s correlation was used to find associations between ocular parameters. There was a positive correlation between AL and both ACD and VD (*P* < 0.001). However, AL was significantly negatively correlated with ACP (*P* < 0.001) (Figure 2). However, ACD had a weak positive correlation with VD (r = 0.151; *P* = 0.017) and no correlation with ACP (*P* > 0.05). ACP was significantly negatively correlated with VD (r = -0.362; *P* < 0.001) but not with ACD (*P* > 0.05) (Table 4).

**Table 4:** Correlations between ocular parameters

Parameter	Correlation	AL	ACD	VD	ACP
AL	*r*	1	0.415	0.788	-0.366
	*P*		<0.001	<0.001	<0.001
ACD	*r*	0.415	1	0.151	0.105
	*P*	<0.001		0.017	0.098
VD	*r*	0.788	0.151	1	-0.362
	*P*	<0.001	0.017		<0.001
ACP	*r*	-0.366	0.105	-0.362	1
	*P*	<0.001	0.098	<0.001	

AL, axial length; ACD, anterior chamber depth; VD, vitreous depth; ACP, anterior corneal power.

## Discussion

This is the first study conducted in Sudan to address the relationship between body and eye parameters in Sudanese adults, aiming to improve the understanding of ocular and systemic diseases for better diagnosis and management. The study demonstrated that age did not influence any ocular or anthropometric parameters (*P* > 0.05). However, significant differences were observed between men and women in age (*P* = 0.42), height (*P* < 0.001), weight (*P* = 0.006) as well as AL (*P* = 0.005) and ACP (*P* < 0.001). No significant differences were found in BMI (*P* = 0.071), ACD (*P* = 0.304), or VD (*P* = 0.141). Body weight and BMI were significantly higher in overweight participants than in normal and underweight participants (*P* < 0.001). However, height and all ocular parameters showed no significant differences across normal weight, underweight, and overweight groups (*P* > 0.05), suggesting that obesity does not have a significant impact on biometric ocular changes. These findings align with studies in the Egyptian adult population, which showed that age was not a risk factor for changes in ocular parameters [1-3]. While body length should stay more or less constant during young adult life, weight and BMI may change, which may explain the lower correlations for weight.

Body height was significantly positively correlated with body weight (r = 0.431; *P* < 0.001), AL (r = 0.340; *P* < 0.001), and VD (r = 0.317; *P* < 0.001). However, body height was significantly negatively correlated with ACPs (r = -0.287; *P* < 0.001). Therefore, taller people tend to have long axial eye lengths, deeper vitreous cavities, and flatter corneas, which compensate for each other and shift the eye toward emmetropia. Body height was not significantly correlated with ACD or BMI (*P* > 0.05). These results are compatible with other studies in Indian and Australian adult populations [6,13].

Body weight showed a significant positive correlation with BMI, AL, and VD (*P* < 0.05), but no significant correlation was found with ACD or ACP. This suggests that individuals with higher body weight tend to have longer axial eye lengths and deeper vitreous cavities, which may predispose them to myopia, although the relationship with myopia was not statistically significant (*P* > 0.05). Additionally, BMI was strongly positively correlated with weight (r = 0.721; *P* < 0.001), but no significant correlations were observed between BMI and other clinical parameters (*P* > 0.05). These findings are consistent with previous studies conducted in Nigerian and Chinese populations, which also reported a higher prevalence of myopia in individuals with obesity [9,14,15].

Furthermore, in vivo ocular measurements demonstrated different correlations with various ocular parameters. There was a significant positive correlation between AL and ACD (r = 0.415; *P* < 0.001) as well as VD (r = 0.788; *P* < 0.001). However, AL was significantly negatively correlated with ACP (r = -0.366; *P* < 0.001), suggesting that longer eyes tend to have deeper anterior chambers, deeper vitreous cavities, and flatter corneas compared to previous studies [1,8,22]. This may decrease the risk of increased intraocular pressure and may prevent glaucoma. Moreover, ACD had a weak positive correlation with VD (r = 0.151; *P* = 0.017) and no correlation with ACP (*P* > 0.05). ACP was significantly negatively correlated with VD (r = -0.362; *P* < 0.001) but not with ACD (*P* > 0.05). These results confirm those of other studies in Singaporean adults, where taller individuals were found to have longer ALs, thinner lenses, deeper ACDs, flatter corneas, and more myopic refraction [18]. Similar trends have been observed in studies on Asian and Malaysian populations, where taller adults had longer ALs and deeper ACDs [19,21]. The results of the current study can help eye care professionals understand the correlation between ocular parameters and body parameters, providing valuable insight for screening, risk assessment, and the diagnosis and treatment of ocular diseases.

A limitation of this study is that it does not provide specific information on the influence of anthropometric measurements on ocular parameters in individuals with obesity. However, one strength of the study is its focus on relatively young, emmetropic subjects, ensuring that the eye and body were well-developed and minimizing the impact of age-related changes.

## Conclusion

This study revealed that taller subjects had significantly longer axial lengths, deeper vitreous cavities, and flatter corneas. Body weight was positively associated with axial eye length and vitreous depth. Body mass index had no effect on any of the biometric ocular parameters.
